# Evaluation of Suitability of New Bedding Material Obtained after Straw Biogasification for Dairy Cows

**DOI:** 10.3390/ani13121905

**Published:** 2023-06-07

**Authors:** Robert Kupczyński, Michał Bednarski, Anna Budny-Walczak, Wojciech Kociuba

**Affiliations:** 1 Department of Environment Hygiene and Animal Welfare, The Faculty of Biology and Animal Science, Wroclaw University of Environmental and Life Sciences, 38c Chelmonskiego St., 50-375 Wroclaw, Poland; anna.budny-walczak@upwr.edu.pl (A.B.-W.); 115256@student.upwr.edu.pl (W.K.); 2 Department of Epizootiology and Clinic of Bird and Exotic Animals, Faculty of Veterinary Medicine, Wroclaw University of Environmental and Life Sciences, 47 Grunwaldzki Sq., 50-366 Wroclaw, Poland; michal.bednarski@upwr.edu.pl

**Keywords:** dairy cow, welfare, preference, bedding material, bacterial count

## Abstract

**Simple Summary:**

Various materials are used for bedding in dairy cow cubicle barns, including straw, sawdust, peat, sand, mats, separated manure, and others. The use of each of these materials requires different financial and labor inputs. Straw is the most commonly used material, characterized by high content of dry matter and the ability to absorb water and gases. A satisfactory bedding material should exhibit a strong physical structure and water absorption capacity to ensure optimal hygienic conditions. The quality of a bedding material deteriorates in direct proportion to the increase in moisture caused by the accumulation of urine and the simultaneous decrease in dry matter. An increase in bedding moisture, temperature, and C/N ratio can promote the growth of bacterial colonies. A sufficient drying capacity and an appropriate pH, which can help to effectively inhibit bacteria, viruses, and fungi, should be ensured when searching for the ideal bedding material. Straw obtained after biogas production can offer an alternative solution for farmers struggling with a shortage of bedding materials.

**Abstract:**

This study aimed to compare the biomass obtained via the biogasification of straw with the classic bedding material, wheat straw. It was divided into two stages. In the first stage, a laboratory evaluation of the bedding materials was carried out, taking into account dry matter, pH, and water absorption. In the laboratory tests, the bedding obtained after the biogasification of straw (Verbio) showed better sorptive properties, with a value of 439.86% (wheat straw’s value was 294.10%), and its pH value was higher than that of wheat straw. In the second stage of the experiment, field tests were carried out on a production farm, wherein the bedding was evaluated for bedding hygiene, animal hygiene, insulation properties, animal productivity, and microbiological properties. A microbiological assessment was also performed. Regarding cleanliness and production parameters and thermographic insulation properties, the two types of bedding did not show statistically significant differences. In terms of microbiological parameters, a higher number of all examined types of bacteria and fungi was observed in the Verbio bedding compared with straw, but these differences were not statistically significant, except in the case of total coliform. The results indicate that straw obtained after gasification is a suitable bedding material, with parameters similar to those of wheat straw.

## 1. Introduction

The bedding material for dairy cow resting areas should meet several criteria. The bedding should be dry, clean, soft, and, at the same time, elastic and not easily distorted, to ensure optimal hygiene conditions and create favorable conditions for cow rest [1,2,3,4]. Bedding materials should provide cows with comfort and facilitate their activities, although the main concerns are the cost and limited supply of bedding materials [5].

Grain straw has thermal insulation and hygroscopic properties, but it has many characteristics that deviate from the ideal bedding material. The parameters of straw, including the short-term sorptive capacity, which necessitates frequent replenishment and leads to greater material consumption, and the variable pH after contact with urine and feces, as well as the maintenance of a high temperature in the bedding, emphasize the need for alternative bedding materials [4]. These factors create conditions that facilitate the development of pathogenic microorganisms [4]. Shane et al. [3] state that a bedding material with a good physical structure, good water absorption capacity, less than 25% initial moisture, and a particle size of less than 2.5 cm is the best option for animal housing systems. The bedding material should minimize the risk of contact between the milk gland ducts and pathogenic microbiota, while guaranteeing a long rest period for cows in their resting areas.

Dairy cows spend 10.0 to 15.5 h lying down [6,7,8]. During this time, bacteria can be transferred from the bedding to the papillary ducts (*ductus papillares*). The dominant view is that the bacteria present in inorganic bedding are usually fewer than those in organic bedding, depending on the bacterial strain and the type of material [8,9]. This is influenced by many factors that are often not directly related to the type of bedding material, e.g., the frequency of changing the bedding. Zdanowicz et al. [9] found that coliforms and Klebsiella spp. on papillary ducts were more numerous when cows were housed on sawdust bedding, but Streptococcus spp. were more numerous on the teats of cows housed on sand. Other studies indicate that different species of bacteria are better controlled when using a compost-bedded pack (CBP) with forest biomass, compared to a CBP with sawdust [8]. On the other hand, cow comfort and wellbeing may be improved when cows are housed in compost-bedded pack barns [7].

The suitability of different bedding materials to improve animal welfare has been studied in terms of lying comfort, the ease of standing up and lying down, and the reduction of the risk of mastitis due to environmental pathogens [7,9,10,11]. There is also great interest in the use of by-products of industry, such as forest biomass [3,8], rice husks and peanut shells [12], recycled sand, digested manure solids [13], and recycling manure solids [14].

This study’s objective was to compare wheat straw, a conventional bedding material, with the biomass produced after biogas production from straw. It was hypothesized that the straw by-product of biogas production would have good physicochemical properties that affect bacterial flora, while providing a bedding material that improves animal comfort.

## 2. Materials and Methods

### 2.1. Laboratory Bedding Material Analysis

This study was conducted in 2020 at the Laboratory of Environmental Hygiene and Animal Welfare, at the Research and Teaching Station in Swojczyce, at the University of Environmental and Life Sciences in Wrocław. This study also involved a cattle farm located in the Lower Silesian Voivodeship in Poland. Verbio was the studied material (VERBIO Polska Sp. z o.o.); it is a treated waste product from the production of second-generation biofuels and is derived from various agricultural raw materials (mainly straw) and residues. After the fermentation process in the biogas facility (by standard conditions), the solid fraction was separated and dried at 90–110 °C. The duration was adjusted to the rate of drying to obtain the desired microbiological purity. The experiment was conducted in two stages, including laboratory tests and field tests.

The laboratory tests aimed to compare the Verbio bedding (V) with wheat straw (WS) and chopped wheat straw (chopped WS, WSch), to create a similar structure to Verbio, with a length of 1.0 mm to 1.7 mm. The dry matter content was determined using a RADWAG WPX 50S laboratory balance (Radwag, Radom, Poland). Approximately 3 g of each test material was placed on the balance pan and dried at a temperature of 130 °C. Three repetitions were performed.

To determine the water absorption capacities, 6 samples of each bedding material, weighing 50 g, were placed in sleeves consisting of a hydrophobic (nylon) material, following the methodology proposed by Teixeira et al. [1]. The samples were then immersed in water for 24 h. After this time, they were removed from the water and hung to drain for 1.5 h. Next, the samples were weighed again, and the water absorption capacities were calculated using the following equation:

$$W=\frac{m_{1}-m_{0}}{m_{0}}\times 100\%$$

where *m*_1_ is the sample mass after soaking [g], and *m*_0_ is the sample mass before immersion [g].

The pH values of the bedding materials were determined in a mixture of distilled water and bedding material (in a 2:1 ratio), using a pH meter with a built-in thermometer (a Mi150 electronic pH meter). Measurements were taken at 0, 0.5, 1, 2, and 24 h and on the 3rd day.

### 2.2. Animals and Experimental Design

**Figure 1:**
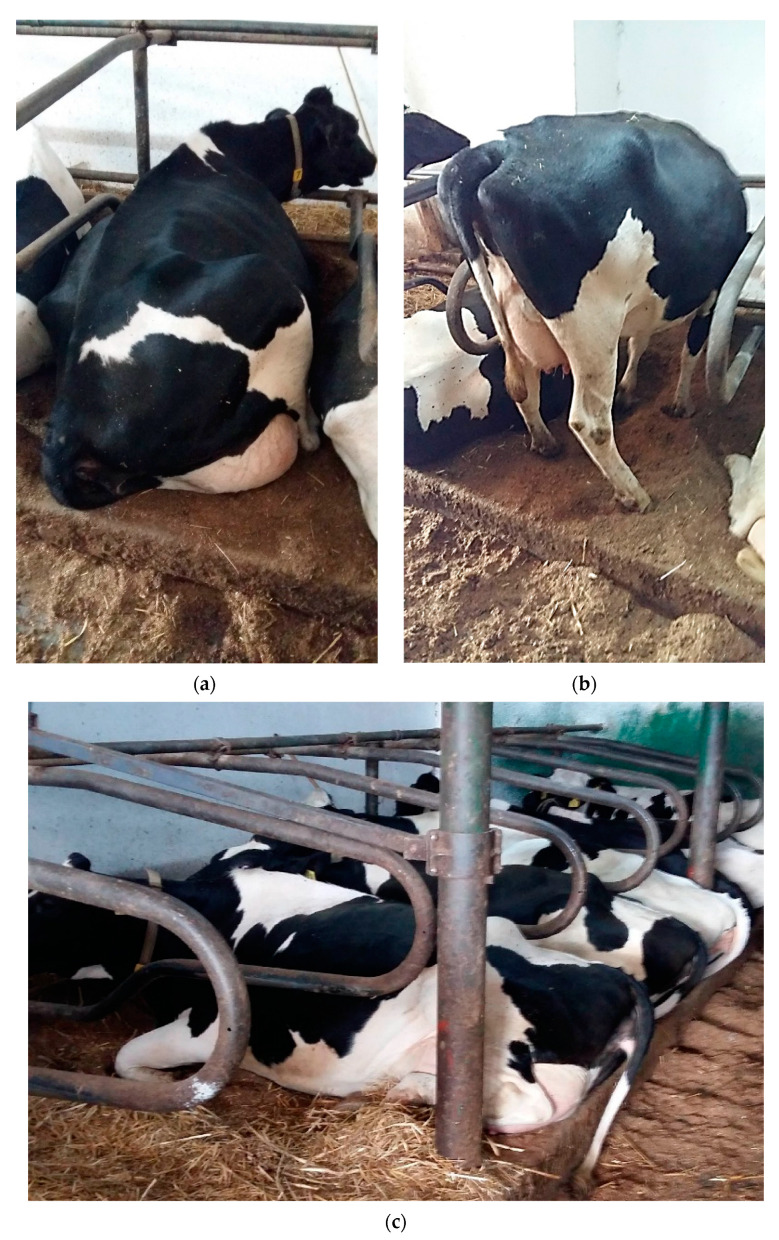
Cow lying on stand with Verbio bedding (**a**). Verbio bedding 3 days after application (**b**). Cows in stalls—Verbio bedding (**c**).

This study was conducted from September to December 2020 in a free-stall barn in a cubicle. The herd consisted of a total of 70 dairy cows with an average lactation yield of 11,000 kg of milk. The cows were monitored for milk productivity. The animals were milked twice a day in a herringbone milking parlor located in the milking hall. The cows were fed according to the TMR method. Fourteen bedding positions were selected for this study, including seven positions lined with wheat straw bedding (WS) and seven positions lined with Verbio bedding (V). The cows participating in this experiment were in their 2nd to 5th lactation. The selected positions for this study were slightly recessed, with dimensions of 210 cm × 120 cm (Figure 1). The selection of positions took into account the constant presence of the same cows. The bedding materials were changed every 7 days, with 13.3–14.0 kg of Verbio bedding and wheat straw used per position, respectively.

To evaluate the visual qualities of the bedding based on dirtiness, moisture, compactness, and homogeneity, a 3-point scale was developed (0—very clean and 3—very dirty; adjusted by 0.5 points). Measurements were taken on the 6th day of the experiment for each tested group. The tests were repeated 3 times, always on the 6th day after applying the bedding material to the stall.

**Figure 2:**
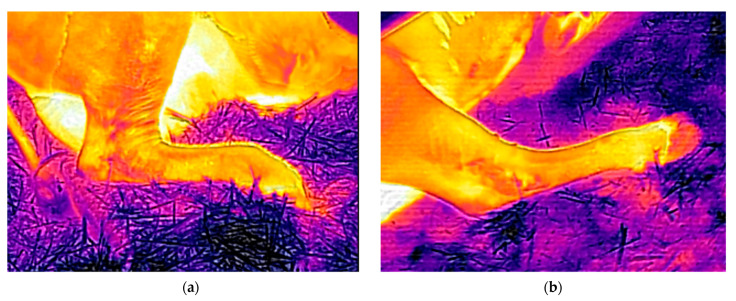
Thermal images showing the distribution of temperature around an animal lying on WS (**a**) and V (**b**) bedding on the 6th day after their application.

In addition, thermal measurements were taken using a Flir E8 thermal imaging camera (Flir Systems AB Company Profile, Täby, Stockholm, Sweden) with emissivity of 0.90. The measurements in the stalls were taken 6 days after applying the bedding materials to the stalls, in 2 repetitions. During the first measurement period, the ambient temperature (T) was 22.6 °C and the relative humidity (f) was 56.2%. In the second repetition, the ambient temperature was 12.2 °C and the relative humidity was 54.8%. Each time, the maximum temperature was recorded immediately after the cow stood up from the bed and again after 15 min (at a distance from the stall of 1.2 m). The thermograms were processed using the FLIR Tools program (version 6.4.18039.1003) (Figure 2). These studies aimed to determine the thermal insulation properties of the stalls and the level of animal comfort.

During the 3-month experimental period, several tests were conducted, including assessments of the cow condition (6 times) and animal cleanliness (10 times) and clinical examinations of hoof health (10 times) and milk yield and composition. The cleanliness of the cows was evaluated based on a 4-point system that considered the following body parts: the abdomen, udders, legs, and pelvic limb hooves. The evaluation was performed using a modified 4-point scale based on the following methodology, proposed by Esser et al. [15]: 1 point—clean, 2 points—slightly dirty, 3 points—dirty, 4 points—very dirty. Hoof health was assessed through visual observation and palpation. The average milk yield and fat, protein, lactose, dry matter, and somatic cell content percentages were calculated based on data from the milk yield evaluation of cattle in Poland (from the RW2 reports in the SYMLEK system, Poland). These parameters were recorded every 4 weeks during this study.

Bedding samples were subjected to microbiological examinations, which included the quantification of the total Gram-negative bacteria count, coliforms, *Klebsiella* sp., *Streptococcus* sp., *Bacillus* sp., fungi, and yeasts. Sample preparation was performed according to the methodologies presented by Hogan and Smith [16] and Zadanowicz et al. [9]. For each sample, inoculations were carried out with ten-fold successive dilutions on appropriate media. Samples were collected from 3 randomly selected sites for each bedding material, using a plastic grid. The edge of the plastic scoop was placed below the surface of the bedding, and the material was collected from a 10 cm layer below the surface. These samples were taken from the bedding site closest to the udder glands, while the cow was lying down. Samples from one bedding site were combined in sterile plastic bags with a capacity of 1 L and transported to the laboratory, where they were examined for the presence of bacteria.

### 2.3. Statistical Analysis

Data were analyzed using the STATISTICA 10.0 software (Statistica, Tulsa, OK, USA). The normality of variables was assessed via the Shapiro–Wilk test. The data on hoof health, bacterial counts, and the final patchiness percentages of the crusted bedding materials were not defined in the statistical model and were thus presented as simple descriptive statistics using only average values. The rest of the animal data were analyzed using the ANOVA procedure, with days as repeated measurements, the treatment as the fixed effect, and the cow as the random effect, where *p*-values were provided. Differences between the mean values of treatment groups were analyzed for significance using the Tukey post hoc test. All are presented as mean values and standard errors of means (SEMs), and significance was defined at *p* ≤ 0.05.

## 3. Results

### 3.1. Laboratory Bedding Material Analysis

The examined bedding materials were characterized by very high content of dry matter (91.4 ± 0.93% for WS, 91.08 ± 0.56% for WSch, and 90.35 ± 0.56% for V). The moisture levels in the bedding materials were 8.58%, 8.60%, and 9.65%, respectively, and these values were confirmed with an organoleptic assessment in which the materials spilled out when held by hand and did not leave any wet imprints.

The range of water absorption capacities varied (Table 1). WS showed the lowest water absorption capacity, while the V bedding showed the highest water absorption capacity. The average of three repetitions was 294.10% for WS, 316.66% for cut WS, and 439.86% for the V bedding.

**Figure 3:**
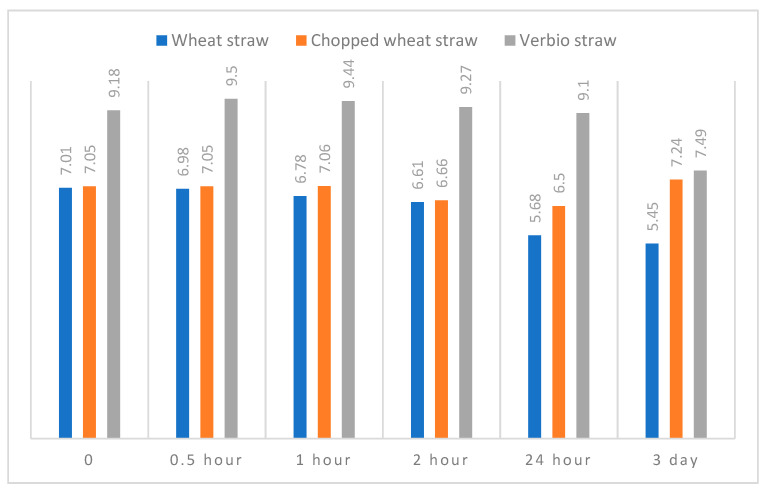
pH values of tested bedding materials in different time intervals.

With the methodology adopted in this research, the pH values of the tested bedding materials were determined after mixing them with distilled water. Wheat straw was characterized by pH values that were very close to neutral (Table 1). A similar pH was determined for WSch. The Verbio litter had an initial pH of 9.18. Over the test period, the pH values of all materials were systematically lowered by 24 h (Figure 3). The smallest decrease in pH at this time was observed for the V bedding (9.10) and the largest was observed for straw (5.68). A further decrease in pH after 3 days was found for the WS and V materials, while the chopped wheat straw (WSch) demonstrated a pH increase of 0.74.

### 3.2. Field Studies on Dairy Cows in a Free-Stall Barn

In the conducted experiment, the litter was changed every 7 days. Its quality was also recorded during this period. This analysis showed that for this type of stand (with a depth of approximately 15 cm, sand at the bottom, and standard dimensions), the weekly litter consumption was 13.3–14.0 kg per stand. After seven days, it was necessary to replace it with a new lot. The observations of the animals showed that they were very willing to use the sites lined with the experimental bedding V. There was no apparent interest in the Verbio bedding as feed in this experiment.

The barn was fully occupied, and the walking corridors were covered with feces for most of the day; thus, feces were carried via the cows’ hooves to the dens. The welfare of the examined cows was in no way impaired by the housing conditions of the animals.

The assessment of the Verbio litter quality after the sixth day of the experiment was the most favorable regarding the following parameters: dirtiness, wetness, and homogeneity (Table 2). The greatest difference between WS and V was noted for the dirtiness parameter (with a 0.67-point difference). In terms of compactness, both bedding materials were rated above two points, but the score was higher for Verbio and amounted to 2.53 points on the three-point scale (Table 2).

The effects of the bedding materials on the cows’ cleanliness scores for individual body parts showed equal values (Table 3). The average cleanliness of individual body parts ranged between clean (score 0) and slightly dirty (score 1), which is considered a very good result for a free-stall barn (without a paddock) with the maximum number of animals. Animals maintained on litter were characterized by lower scores in terms of abdomen, leg, and feet cleanliness. However, the evaluations of udder cleanliness were equal. The largest difference was noted for the pelvic limb hoof cleanliness parameter, which was 1.47 for WS and 1.69 for V (with a difference of 0.22). In general, the skin in these places was most exposed to dirt as a result of constant contact with the ground, which was stained with feces and urine. Animals also moved on the litter with different velocities.

**Figure 4:**
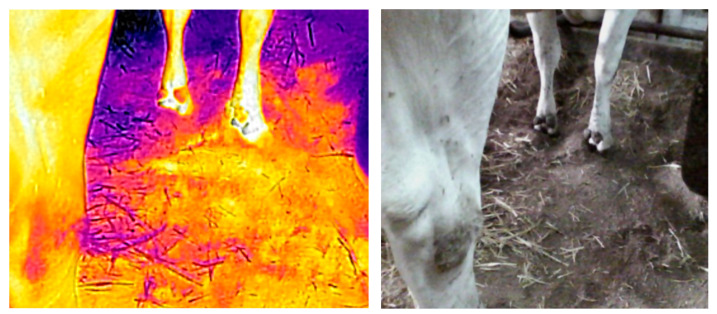
Infrared illustration of Verbio bedding material (**left**) and normal photograph (**right**) after standing up. Maximum temperature of the litter was 33.2 °C (even temperature distribution).

**Figure 5:**
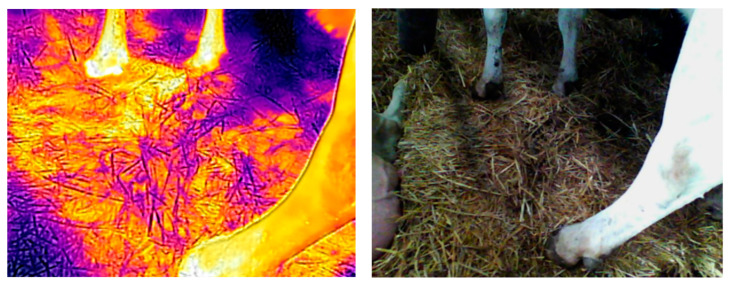
Infrared illustration of wheat straw bedding (**left**) and normal photograph (**right**) after standing up. Maximum temperature of the litter was 32.6 °C (uneven temperature distribution).

The measurements with the thermal imaging camera did not show significant differences in the stalls’ surface temperatures (Table 4). Regardless of the measurement date, the temperature immediately after the animals stood up from the ground was equalized between the groups. The animals were more willing to use and remained for longer on the V bedding, which also had a lower ambient temperature (12 °C). The temperature of the V bedding after the animals stood up was slightly higher (30.68 °C and 30.02 °C). The Verbio bedding material was characterized by a higher heat capacity (which depended on the mass and density of the material) compared with straw. When the cows were lying on the Verbio bedding on the sixth day after application, the area around the animals had a higher temperature, and its range was greater (Figure 2, Figure 4 and Figure 5). Lower bedding temperatures were recorded for the V litter—23.11 °C and 19.93 °C in individual repetitions—15 min after the cows stood up. The temperature distribution in the Verbio litter was more even than that in the wheat straw (Figure 4 and Figure 5).

The average milk yields for the entire study period did not show statistically significant differences between the groups (Table 5). Higher fat content was recorded in the Verbio group and amounted to 3.97%, while the protein content compared with the control group was slightly lower, at 3.28%. The somatic cell count (SCC) in the Verbio group was 92.38 × 1000 cells/mL and was higher than in the control group. However, the observed SCC values indicated very good quality of milk. No inflammation in the mammary glands was noted during the study period. The bacterial counts (BCs) were equal in both treatment groups (3.29 × 1000 IBC/mL and 3.30 × 1000 IBC/mL).

The numbers of bacteria in the WS bedding compared with those in the V bedding are presented in Table 6. On the seventh day of use of the bedding materials, higher numbers of all the examined types of bacteria and fungi were observed in the Verbio bedding compared with wheat straw, but these differences were not statistically significant. The total Gram-negative bacteria counts were 6.4 log10 cfu/g for WS and 6.8 log10 cfu/g for V, and the *Klebsiella* counts were 4.4 log10 cfu/g and 4.6 log10 cfu/g, respectively. Streptococci were the most numerous of the examined bacteria, with their numbers amounting to 7.7 log10 cfu/g in the classic bedding and 7.9 log10 cfu/g in the V bedding. In the case of fungi, the numbers were as follows: 0.2 and 0.7 log10 cfu/g for yeast in the WS and V bedding, and 1.3 cfu/g and 1.9 cfu/g for molds, respectively. The dominant species of fungus was *Aspergillus niger*.

## 4. Discussion

Straw is the main material used for the housing of cows in many countries. To determine the reasons behind this, two factors can be considered: the low cost and the widespread availability of the raw material. The quality of the bedding deteriorates in direct proportion to the increase in humidity caused by the accumulation of urine and the simultaneous decrease in dry matter content. Bedding layered with straw can create conditions conducive to the development and survival of various bacterial species, including those involved in mastitis etiology [10]. Additionally, manure-contaminated straw bedding maintains high humidity, and the pH of clean straw, which is neutral, quickly drops and remains at a pH level of 5.5 to 6.5 after contamination. The rapid proliferation of pathogens in such conditions is an indirect factor in the occurrence of udder and hoof diseases and also results in economic losses in livestock farming [10]. When searching for an ideal bedding material, efforts should be made to achieve an alkaline substrate pH value that remains at a level of 9.0 and inhibits the growth of unwanted bacteria [17]. Meng et al. [18] indicate a relationship between high pH values and the ammonia content in bedding. They explain that high pH values in bedding promote good permeability for ammonia [18].

Dunlop et al. [4] propose that the ideal bedding material is dry, absorbs moisture well but dries easily, and allows animals to exhibit their natural behavior. In our studies comparing wheat straw and Verbio bedding, which was a form of treated waste from biofuel production and whose appearance resembled brown chaff, the Verbio bedding was characterized by high dry matter content, a pH above neutral, and a high water absorption capacity that was significantly higher than that of wheat straw. The values for chopped wheat straw (WSch) were higher in this experiment than those reported by Wolfe et al. [19] for an analogous bedding material. Wolfe et al. [19] also indicated that the highest DM content in the tested bedding materials was 89.8%. These are higher results than those obtained by Li et al. [12] for peanut shell, a peanut–rice combination, and rice husk, which were 83.8, 82.5, and 82.7%, respectively. In [20], bedding samples with higher DM content had the lowest levels of bacterial growth compared to those with lower DM content.

The high pH value in the Verbio bedding after contact with water may have been related to the bacteriostatic action of this material. High water absorption, i.e., the absorption and storage of water, is a desirable property in bedding materials according to Dunlop et al. [4]. The greatest differences were in terms of dirtiness, wetness, and homogeneity. Teixeira et al. [1] obtained a better overall result for wheat straw bedding but worse parameters, such as dirtiness and humidity. The difference in results could be related to the different lengths of time for which the animals used the bedding.

Monitoring the surface temperature of litter in composting facilities is an important parameter in verifying its quality [21]. The temperature of a bedding material depends on the air temperature, the type of material, and its depth [18]. The experiments using thermographic imaging showed an even temperature distribution in the Verbio bedding. While an animal was lying down, the area around it had a higher temperature, and its range was greater, at 30.68 and 30.02 °C in the first and third months of measurement. Meng et al. [18] obtained similar temperatures, between 24 and 30 °C, from their measurements of experimental litters that consisted of a mixture of different materials. Biasato et al. [22] described a compost-bedded pack barn bedding material with a mean temperature of 31.02 ± 1.57 °C.

The experimental bedding lost more heat than the WS bedding 15 min after the animals woke up. These results indicated a higher water capacity, which was also demonstrated in in vitro tests. Moist surfaces lost more heat per unit of time due to evaporation. The deep straw bedding showed higher thermal insulation and hygroscopic properties, as well as constant microbiological processes within it. Moreover, in the study by Borshch et al. [23], it also showed higher temperatures when cows were lying down.

The average cleanliness scores of the individual body parts of high-yielding cows maintained on Verbio bedding ranged from clean to slightly dirty (scores of 0–1). Similar results were reported by Wolfe et al. [19]. These cleanliness scores should be considered very good for a free-stall barn with full animal occupancy. The degree of soiling on the udders, pelvic limb hooves, and abdomen did not have a significant impact on the milk quality or on the occurrence of mastitis. However, in terms of the health status of hooves, the bedding materials that were used had similar effects, and no lameness was observed, which largely resulted from the properly conducted preventive measures to maintain the hooves in the herd. Li [12] obtained similar results for udder cleanliness on peanut shell bedding (with scores of 1.8 to 1.83 for both types of bedding), as did Esser et al. [15]. Interestingly, in Esser et al.’s [15] study, leg cleanliness was very poor for all the tested bedding materials, with scores of over 2.2, compared to the much lower values found in the present study. Cows maintained in farms with mattress-based stalls had a higher prevalence of dirty udders compared to those using a deep bedding system [24]. Additionally, these studies indicated that wider stalls were associated with a lower bulk milk SCC and bacterial count. High numbers of bacteria were observed both within and between the bedding samples in [20]. This variability may overshadow any potential associations between milk quality and the type of bedding.

In our research, the average milk yield was 38.20 kg for WS bedding and 38.63 kg for V bedding. Rowbotham and Ruegg [25] found that cows in their first lactation produced 33.20 kg when using new sand bedding, 30.20 kg when using recycled sand, 30.70 kg when using deep bedding, and 30.40 kg when using shallow bedding. In the study by Esser et al. [15], milk production ranged from 33.50 to 34.40 kg, with the highest yield of 34.40 kg produced with deep-bedded organic solids. The highest fat content was produced with the V bedding at 3.97%, while WS bedding produced the highest protein content at 3.46%. In the study by Esser et al. [15], the fat content when using all types of bedding was at the same level of 3.90–3.92%, while the protein content ranged from 3.12 to 3.17%. Due to the lack of precise data on animal nutrition in other studies, it is difficult to compare their milk production. In our research, the nutritional factor was constant and not related to changes in milk composition [26], and the environmental factor was not associated with suboptimal behavior among animals [27].

The bacterial concentrations in bedding are strongly linked to the cows’ hygiene and milk quality. In the study by Esser et al. [15], mastitis was detected in nine cows (out of 62) housed on sand bedding and in seven (out of 60) housed on recycled sand, which is a large difference compared to the results of the study by Rowbotham and Ruegg [25], who detected 15 and 26 cases of mastitis when using the same bedding materials, respectively. Rowbotham and Ruegg [25] evaluated bedding consisting of undigested plant parts recovered from manure and MAT (the same bedding, but lined with mattresses) and found 23 (out of 62) and 15 (out of 63) cases of mastitis, respectively. The type of bedding is associated with the bacterial count, which is associated with the prevalence of diseases such as mastitis [24]. Organic bedding was previously found to be less contaminated than inorganic materials [9,13,24,28]. The total bacterial count in the Verbio bedding was similar to the value in the control bedding material. There were also no statistically significant differences in the range of bacterial types, such as Streptococcus or Klebsiella. The number of bacteria in the bedding is also related to the percentage of dry matter. In the present study, the dry matter in both bedding materials was similar, which may explain the similar microbiological results.

The microbiological results presented in this study were similar to or lower than those obtained in other studies [9,13]. The results of this study indicated that the Verbio bedding did not differ significantly from other straw-based bedding materials. Although there was a lack of statistical differences, higher numbers of total Gram-negative bacteria, yeast, and fungi were found in the Verbio bedding after its application in the cow stalls. Structural changes and increased porosity during the material’s bioprocessing increased its sorption capacity. However, this also likely enabled the growth of microorganisms due to an increase in pores. Other studies have indicated such a relationship [29]. In one study, the total coliform, *Escherichia coli*, and *Staphylococcus aureus* counts in sawdust were higher than in forest biomass in period 1 but did not differ between treatments in period 2. This indicated a large change in dynamics in the microorganisms in the bedding, depending on various factors [8]. In other studies, environmental mastitis pathogens were isolated from bedding materials, soil, rumen, feces, vulva, and feed samples, indicating a risk of environmental contamination and papillary duct infection. In our work, the number of environmental bacteria was similar in both bedding materials [20,30]. Generally, bedding management can have a significant impact on milk quality, the bacterial concentrations in bedding, and cow hygiene.

## 5. Conclusions

Based on the obtained results, Verbio bedding can be used as the primary bedding material for the maintenance of dairy cows in a bedding system. This bedding material can serve as an alternative to wheat straw, with similar physical properties. In free-stall barns, it needs to be replaced every 6 days. In the study, dairy cows were very willing to lie on the Verbio bedding, without consuming it as feed. It has good water absorption properties and does not cause excessive bacterial growth. In future studies on the use of Verbio bedding, it will be necessary to evaluate the time and costs associated with cows lying on this material, thus determining their comfort and welfare. Long-term studies are required regarding the health of cows’ mammary glands and hooves.

## Figures and Tables

**Table 1:** Average dry matter (DM) content and assessment of water absorption capacities of WS, WSch, and V bedding (means, SEM).

Item	WS	WSch	V	SEM
DM (%)	91.08	91.40	90.35	8.54
M (%)	8.58	8.60	9.65	2.87
W (%)	294.10	313.66	439.86	14.32
Initial pH	7.01	7.05	9.18	0.86

WS—wheat straw control group; WSch—chopped wheat straw; V—Verbio experimental group; DM—dry matter; M—moisture; W—water absorption.

**Table 2:** Effects of treatments on bedding quality scores (means, SEM).

Item ^a^	WS	V	SEM	*p*-Value		
				T	P	T × P
Dirtiness	1.99	1.32	1.32	0.042	NS	NS
Wetness	1.93	1.47	1.43	0.051	NS	NS
Compactness	2.41	2.53	1.49	NS	NS	NS
Homogeneity	1.51	0.98	1.03	0.034	NS	NS
Overall score	1.96	1.57	1.16	NS	NS	NS

^a^ Score (0–3); WS—wheat straw control group; V—Verbio experimental group; T—treatment effect; P—period effect; T × P—treatment × period interaction effect; NS—not significant.

**Table 3:** Effects of bedding materials on the cleanliness scores of cows (means, SEM).

Item ^a^	WS	V	SEM	*p*-Value		
				T	P	T × P
Abdomen	1.29	1.36	0.87	0.054	NS	NS
Udder	1.83	1.83	1.23	NS	NS	NS
Leg	1.53	1.57	0.94	NS	NS	NS
Pelvic limb hoof	1.47	1.69	0.98	NS	NS	NS

^a^ Score (0–3); WS—wheat straw control group; V—Verbio experimental group; T—treatment effect; P—period effect; T × P—treatment × period interaction effect; NS—not significant.

**Table 4:** Average maximum temperatures of the bedding surfaces from thermovision measurements (°C).

Time	WS		V		SEM	*p*-Value	
	0	15 min	0	15 min		T	TP
1 month (°C) (air T = 22.6 °C; f = 56.2%)	30.10 ^A^	23.41 ^B^	30.68 ^A^	23.11 ^B^	0.69	NS	NS
3 months (°C) (air T = 12.2 °C; f = 54.8%)	29.50 ^A^	20.35 ^B^	30.02 ^A^	19.93 ^B^	0.91	NS	NS

^A,B^ Mean values with different superscripts within a row differ (Tukey adjusted *p* < 0.01). WS—wheat straw control group; V—Verbio experimental group; T—treatment effect; TP—time effect; NS—not significant.

**Table 5:** Effects of bedding materials on milk.

Item	WS	V	SEM	*p*-Value		
				T	P	T × P
Milk per cow (kg)	38.20	38.63	8.67	NS	NS	NS
Fat (%)	3.82	3.97	0.65	NS	NS	NS
Protein (%)	3.46	3.28	0.45	NS	NS	NS
SCC (×1000 cell/mL)	87.63	92.38	10.87	NS	NS	NS
BCs (×1000 IBC/mL)	3.29	3.30	0.34	NS	NS	NS

WS—wheat straw control group; V—Verbio experimental group; SCC—somatic cell count; BCs—bacterial counts; T—treatment effect; P—period effect; T × P—treatment × period interaction effect; NS—not significant.

**Table 6:** Microorganism counts (log10 cfu/g) resulting from microbial cultures in raw bedding materials.

Microorganism Count (log10 cfu/g)	WS	V	SEM	*p*-Value		
				T	P	T × P
Total Gram-negative	6.4	6.8	1.46	NS	0.05	NS
Total coliforms	5.3	5.9	0.13	0.03	0.07	NS
*Klebsiella* sp.	4.4	4.6	0.12	NS	NS	NS
*Streptococcus*	7.7	7.9	0.09	NS	0.09	NS
*Bacillus*	2.7	2.8	0.21	NS	NS	NS
Yeast	0.2	0.5	0.07	0.06	0.02	0.01
Fungi	1.3	1.9	0.17	0.09	0.01	0.01

WS—wheat straw control group; V—Verbio experimental group; T—treatment effect; P—period effect; T × P—treatment × period interaction effect; NS—not significant.

## Data Availability

The data are available from the corresponding authors.
